# Diet Quality as a Mediator Linking Tooth Loss and Cardiovascular Diseases in Older Adults

**DOI:** 10.1111/jre.70080

**Published:** 2026-02-26

**Authors:** Ashish C. Kalhan, Tosha A. Kalhan, Marco A. Peres, Fábio R. Leite, Gustavo G. Nascimento

**Affiliations:** ^1^ National Dental Research Institute Singapore, National Dental Centre Singapore Singapore Singapore; ^2^ Oral Health Academic Clinical Programme, Duke‐NUS Medical School Singapore Singapore; ^3^ School of Dentistry University of Utah Salt Lake Utah USA

**Keywords:** cardiovascular diseases, diet quality, healthy eating index, tooth loss

## Abstract

Drawing on a large, nationally representative population of older American adults (*n* = 3610), the study shows that individuals with more than eight missing teeth have a 6%–10% higher likelihood of cardiovascular disease compared with those with fewer tooth losses. Poorer diet quality emerged as a key pathway linking tooth loss and cardiovascular outcomes.

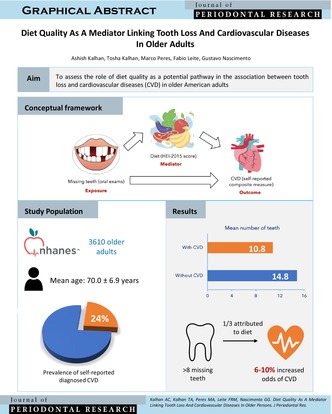

## 1 Introduction

Cardiovascular disease (CVD) remains the world's leading cause of death, responsible for nearly 20 million deaths in 2019 and projected to reach 24 million by 2030 [1, 2]. The growing burden is largely driven by population aging, especially in low‐ and middle‐income countries where healthcare resources are limited. Edentulism, often resulting from untreated caries and periodontitis and affecting 353 million people worldwide [3], is highly prevalent in older persons and represents major contributors to oral health–related disability. Although tooth loss has been linked to various CVD outcomes [4], underlying mechanisms remain unclear.

Tooth loss impairs masticatory function, leading to poorer diet quality, characterized by reduced intake of fruits, vegetables, and high‐fiber foods and greater dependence on processed, calorie‐dense options [5]. These dietary shifts can worsen metabolic health and elevate CVD risk, whereas adherence to high‐quality dietary patterns is associated with lower CVD mortality [6]. We have recently shown population‐level impact of tooth preservation on diet quality using plausible policy‐related emulated scenarios [7]. Building on this, the present study explores if diet quality may constitute a key behavioral pathway linking tooth loss and cardiovascular outcomes.

## 2 Methods

Data were obtained from the 2011–2012 and 2013–2014 cycles of the National Health and Nutrition Examination Survey (NHANES), a nationally representative, population‐based survey of non‐institutionalized U.S. civilians based on a stratified, multistage probability sample. Detailed survey design, interview, and examination protocols have been published elsewhere [8]. Of the 19 931 participants across both cycles, our analyses were restricted to adults aged 60 years and older who completed periodontal examinations, to focus on a population with a higher prevalence of both tooth loss and CVD, thus improving statistical power and relevance for detecting meaningful associations (please refer to the Graphical Abstract).

The exposure measure was the number of missing teeth, assessed through standardized dental examinations conducted by calibrated dentists who recorded the number of permanent teeth present in both arches, which was categorized as > 8 missing teeth versus ≤ 8 missing teeth, corresponding to the absence versus presence of functional dentition. The outcome, CVD, was defined using self‐reported diagnoses of coronary heart disease, congestive heart failure, angina, heart attack, or stroke, collected through medical questionnaires. Diet quality, the presumed mediator, was assessed using the Healthy Eating Index (HEI)‐2015, derived from food‐frequency questionnaires, 24‐h dietary recalls, and supplement intake, which reflect adherence to the Dietary Guidelines for Americans and encompass adequacy (e.g., fruits, vegetables, whole grains, plant proteins) and moderation (e.g., sodium, added sugars, saturated fats) components [9]. Confounders (sex, race, poverty‐income ratio, smoking status, diabetes and presence of dental prosthesis) were identified a priori and self‐reported via structured interviews. Parametric g‐formula assessed diet quality as an exploratory pathway in the tooth loss‐CVD association, decomposing total effects into natural direct and indirect effects, with the proportion of indirect effect quantifying diet's contribution (Graphical Abstract). Standard errors and confidence intervals were estimated using 1000 non‐parametric bootstrap samples.

Sensitivity analyses included adjusting the model for additional covariates (age, education, body mass index, physical activity, glycated hemoglobin, lipid profiles) and handling missing data via Multiple Imputation by Chained Equations. *E*‐values quantified robustness to unmeasured confounding. All analyses accounted for NHANES survey weights, strata, and primary sampling units (PSU). In the g‐formula, weights were applied when estimating conditional outcome and covariate models, ensuring counterfactual estimates reflected the survey design. Multiple imputation was conducted within this framework, with results pooled using Rubin's rules. Variance estimates combined survey‐design–adjusted standard errors with between‐ and within‐imputation variability to capture both sampling complexity and missing‐data uncertainty. Analyses were conducted in Stata 18 (StataCorp, College Station, TX, USA).

## 3 Results

A total of 3610 participants were included (mean age: 70.05 ± 6.97 years), with 23.7% reported as having been diagnosed with CVD. Participant characteristics with respect to the outcome are described in Table S1. The findings showed that having more than 8 missing teeth was associated with a 6%–10% increased odds of CVD compared with having eight or fewer missing teeth, with approximately one‐third of the association attributed to diet quality (Table 1). Sensitivity analyses yielded consistent results, with *E*‐values (1.25–1.49) indicating modest robustness to unmeasured confounding.

**TABLE 1:** Role of diet quality as a potential pathway in the association of tooth loss and cardiovascular diseases in older American persons.

		Outcome: presence of CVD								
		CDE		NDE		NIE		TE		PM^a^
Exposure	Mediator	OR (95% CI)	*p*	OR (95% CI)	*p*	OR (95% CI)	*p*	OR (95% CI)	*p*	%
*Complete case analysis* ^b^ *(n = 2448)*										
> 8 missing teeth^c^	HEI‐2015	1.07 (1.03–1.11)	< 0.001	1.06 (1.02–1.09)	< 0.001	1.04 (1.01–1.08)	0.014	1.10 (1.07–1.14)	< 0.001	42%
*Sensitivity analysis #1: Adjusting for additional relevant covariates* ^d^ *(n = 1108)*										
> 8 missing teeth^c^	HEI‐2015	1.09 (1.04–1.13)	< 0.001	1.05 (1.01–1.09)	0.002	1.02 (0.83–1.05)	0.33	1.07 (1.02–1.11)	0.002	27%
*Sensitivity analysis #2: Accounting for missing data* ^e^ *(n = 3470)*										
> 8 missing teeth^c^	HEI‐2015	1.07 (1.03–1.11)	0.001	1.04 (1.01–1.08)	0.026	1.02 (0.98–1.05)	0.31	1.06 (1.02–1.09)	0.002	25%

*Note:* Point estimates and 95% CI were estimated by the parametric g‐formula in 1000 bootstrapped datasets.

Abbreviations: BMI, body mass index; CDE, controlled direct effect; CI, confidence interval; CVD, cardiovascular disease; HbA1c, glycated hemoglobin; HDL, high‐density lipoprotein; HEI‐2015, healthy eating index; LDL, low‐density lipoprotein; NDE, natural direct effect; NIE, natural indirect effect; OR, odds ratio; PM, proportion mediated; TE, total effect.

^a^ Proportion mediated (PM) is calculated as estimates of NIE/TCE.

^b^ Adjusted for sex, race, poverty‐income ratio, smoking status, self‐report of diabetes, and use of dental prosthesis.

^c^ Reference group: ≤ 8 missing teeth (equivalent to presence of functional dentition, i.e., > 20 natural teeth present).

^d^ Adjusted for additional relevant covariates (age, education, BMI, physical activity, HbA1c, HDL, and LDL).

^e^ Missing data were handled using multiple imputation using the chained equation (MICE) method.

## 4 Conclusions

To the best of our knowledge, this is the first study exploring diet quality as a potentially modifiable pathway linking tooth loss to cardiovascular disease (CVD) in older individuals, leveraging a large, nationally representative dataset of US adults. However, findings should be cautiously interpreted owing to a cross‐sectional study design, limiting the ability to rule out reverse causation, residual confounding, and full adherence to mediation assumptions, and use of self‐reported data for mediator and outcome, potentially introducing bias. Although microbiome data were not available, the oral‐gut microbiome axis may represent a key mechanistic link between tooth loss, diet quality, and cardiovascular risk. These findings underscore the need for further longitudinal and interventional research to clarify whether tooth preservation can prevent adverse dietary changes and downstream cardiometabolic consequences.

## Author Contributions

**Ashish C. Kalhan:** conception, design, data analysis, interpretation, drafting of the work and critical review. **Tosha A. Kalhan:** design, analysis, interpretation, and critical review. **Marco A. Peres:** design, interpretation, and critical review. **Fábio R. Leite:** design, interpretation, and critical review. **Gustavo G. Nascimento:** conception, design, analysis, interpretation, and critical review. All authors gave their final approval and agreed to be accountable for all aspects of the work.

## Ethics Statement

National Center for Health Statistics (NCHS) Ethics Review Board (ERB) #2011‐17, #2018‐01.

## Conflicts of Interest

The authors declare no conflicts of interest.

## Supporting information


**Table S1:** Participant characteristics of 3610 older American persons (aged ≥ 60 years) with and without cardiovascular diseases (NHANES, 2011–2014).

## Data Availability

The data that support the findings of this study are openly available in the National Centre for Health Statistics, Centers for Disease Control and Prevention, at https://wwwn.cdc.gov/nchs/nhanes/.
