# Supplementary material for: Diet Quality as a Mediator Linking Tooth Loss and Cardiovascular Diseases in Older Adults
Source: J Periodontal Res. 2026 Feb 26;61(5):627–9. doi: 10.1111/jre.70080 (PMC13378183; doi:10.1111/jre.70080)
Supplement: Supplementary file 1 — Table S1: Participant characteristics of 3610 older American persons (aged ≥ 60 years) with and without cardiovascular diseases (NHANES, 2011–2014). [file JRE-61-627-s001.docx]

Table 1. Participant Characteristics of 3,610 older American persons (aged >=60 years) with and without cardiovascular diseases (NHANES, 2011-2014)

| Variables | **Presence of CVD**  **n = 857** | | **No CVD**  **n = 2753** | |  |
| --- | --- | --- | --- | --- | --- |
|  | N | % (95% CI)^1^ | N | % (95% CI)^1^ | p-value^2^ |
| ***Number of missing teeth*** |  |  |  |  | <0.001 |
| >8 missing teeth | 375 | 51.9 (45.9-57.7) | 1000 | 35.0 (31.0-39.2) |  |
| <=8 missing teeth | 226 | 48.1 (42.2-54.0) | 1195 | 65.0 (60.7-68.9) |  |
| Missing | 256 | - | 558 | - |  |
| ***Diet quality (HEI-2015)*** |  |  |  |  | 0.001 |
| Poor (<54.3) | 410 | 53.4 (46.9-59.8) | 1151 | 48.9 (44.5-53.4) |  |
| Good (>=54.3) | 318 | 46.6 (40.1-53.1) | 1172 | 51.1 (46.6-55.5) |  |
| Missing | 129 | - | 430 | - |  |
| ***Sex*** |  |  |  |  | <0.001 |
| Male | 465 | 53.1 (48.2-57.9) | 1285 | 42.7 (40.6-44.8) |  |
| Female | 392 | 46.9 (42.0-51.7) | 1468 | 57.2 (55.1-59.3) |  |
| ***Race*** | | | | | <0.001 |
| Mexican American | 55 | 2.9 (1.6-5.3) | 281 | 4.1 (2.6-6.3) |  |
| Other Hispanics | 63 | 3.0 (1.8-4.9) | 300 | 4.1 (2.7-5.9) |  |
| Non-Hispanic White | 459 | 78.5 (74.7-81.8) | 1179 | 77.1 (72.4-81.1) |  |
| Non-Hispanic Black | 200 | 9.4 (7.2-12.0) | 666 | 8.9 (6.6-11.8) |  |
| Others | 80 | 6.2 (4.3-8.6) | 327 | 5.8 (4.5-7.3) |  |
| ***Poverty-income ratio (PIR)*** | | | | | <0.001 |
| 0 - 1.08 | 230 | 17.2 (13.8-21.2) | 544 | 12.1 (10.3-14.1) |  |
| 1.09 - 2.81 | 332 | 44.3 (40.0-48.7) | 948 | 32.8 (28.8-36.9) |  |
| 2.82 - 5.0 | 223 | 38.5 (33.0-44.1) | 993 | 55.1 (50.0-60.1) |  |
| Missing | 72 | - | 268 | - |  |
| ***Smoking status*** | | | | | <0.001 |
| Current smoker | 132 | 14.3 (11.1-18.3) | 332 | 10.4 (8.6-12.4) |  |
| Former smoker | 360 | 44.4 (40.4-48.5) | 980 | 37.1 (33.9-40.5) |  |
| Never smoker | 365 | 41.3 (36.8-45.7) | 1437 | 52.5 (49.2-55.6) |  |
| Missing | - | - | 4 | - |  |
| ***Self-report of diabetes*** | | | | | <0.001 |
| Yes | 314 | 34.0 (30.5-37.7) | 552 | 16.7 (14.8-18.8) |  |
| No | 500 | 66.0 (62.2-69.4) | 2085 | 83.3 (81.2-85.1) |  |
| Missing | 43 | - | 116 | - |  |
|  |  | Mean (SD) |  | Mean (SD) |  |
| **Number of missing teeth** | 601 | 14.8 (10.6) | 2195 | 10.8 (10.1) | <0.001 |
| **HEI-2015** | 728 | 52.8 (13.3) | 1490 | 54.8 (13.9) | 0.21 |
| **Age** | 857 | 72.5 (6.8) | 2753 | 69.2 (6.8) | <0.001 |

^1^ Weighted percent and 95% CI provided by NCHS. ^2^ p-values are based on the Rao-Scott correction for weighted chi-square test.

Abbreviations: CVD, cardiovascular diseases *(including self-reported events of coronary heart disease, congestive heart failure, angina pectoris, heart attack and stroke);* CI, confidence interval.
